# Monocular Vision System for Fixed Altitude Flight of Unmanned Aerial Vehicles

**DOI:** 10.3390/s150716848

**Published:** 2015-07-13

**Authors:** Kuo-Lung Huang, Chung-Cheng Chiu, Sheng-Yi Chiu, Yao-Jen Teng, Shu-Sheng Hao

**Affiliations:** 1 Department of Electrical and Electronic Engineering, Chung Cheng Institute of Technology, National Defense University, Taoyuan 33551, Taiwan; E-Mails: 1040510304@ndu.edu.tw (K.-L.H.); 1030510306@ndu.edu.tw (S.-Y.C.); shushenghao@gmail.com (S.-S.H.); 2 School of Defense Science, Chung Cheng Institute of Technology, National Defense University, Taoyuan 33551, Taiwan; E-Mail: c31020410@ndu.edu.tw

**Keywords:** unmanned aerial vehicle (UAV), monocular camera, stereo vision, motion vector detection

## Abstract

The fastest and most economical method of acquiring terrain images is aerial photography. The use of unmanned aerial vehicles (UAVs) has been investigated for this task. However, UAVs present a range of challenges such as flight altitude maintenance. This paper reports a method that combines skyline detection with a stereo vision algorithm to enable the flight altitude of UAVs to be maintained. A monocular camera is mounted on the downside of the aircraft’s nose to collect continuous ground images, and the relative altitude is obtained via a stereo vision algorithm from the velocity of the UAV. Image detection is used to obtain terrain images, and to measure the relative altitude from the ground to the UAV. The UAV flight system can be set to fly at a fixed and relatively low altitude to obtain the same resolution of ground images. A forward-looking camera is mounted on the upside of the aircraft’s nose. In combination with the skyline detection algorithm, this helps the aircraft to maintain a stable flight pattern. Experimental results show that the proposed system enables UAVs to obtain terrain images at constant resolution, and to detect the relative altitude along the flight path.

## 1. Introduction

The images captured by satellites are important in application areas such as measurement [1], disaster regions [2], agricultural surveys [3], and river monitoring [4]. However, these images are limited by the time at which the satellites pass overhead, occlusion by clouds, and cost. Consequently, this method is not able to capture remote sensing images that completely satisfy the needs of customers. Further, clear images cannot be obtained on cloudy days. Unmanned aerial vehicles (UAVs) have the advantages of mobility, altitude control, and low cost. Using camera-equipped UAVs to capture images, the time, cloud occlusion, and cost issues experienced by satellites can be overcome.

UAVs equipped with cameras are relatively inexpensive, as the cameras supplied with commercial products are limited to a fixed focus; as a result, they cannot capture clear images at different altitudes. Therefore, the UAVs need to fly at a fixed distance from the ground to capture clear images. Hence, maintaining a steady altitude is an important issue for UAVs. Various sensors can help to detect the altitude. In recent research on distance measurement situations such as this, Nagai *et al.* [5] proposed the use of lasers to provide 3-D point cloud data, and Lin *et al.* [6] developed a method that uses light detection and ranging (LIDAR) and real-measured point clouds to estimate height. The laser or LIDAR sensors obtain distance information very simply and quickly but increase equipment costs and payload. A small scale LIDAR, for e.g., URG-04LX-UG01, weighs around 160 g and its effective detection distance ranges from 0.02 m to 5.6 m. Because the flight altitude of this study is around 30 m, the small scale LIDAR is not suitable to detect the relative altitude. These sensors are not suitable for application to small UAVs.

Another method of measuring altitude involves vision sensor systems. This has the advantages of low payload and low cost. Weiss *et al.* [7] used a monocular simultaneous localization and mapping (SLAM) framework to detect the posture of a micro helicopter to perform the basic maneuvers such as hovering, set point and trajectory following, vertical takeoff, and landing. Li *et al.* [8] used a parallel tracking and mapping (PTAM) algorithm to estimate the attitude angle of a helicopter for providing a solution to the trajectory-tracking problem. These algorithms can detect the attitude of a helicopter but cannot detect the relative altitude. Cherian *et al.* [9] proposed an image detection algorithm that can measure the altitude of a UAV using a single camera. The method applies semi-supervised machine learning to images captured from different altitudes. The learned texture information is then used to recognize the input image and determine the altitude. Because the altitude measurement depends on image recognition, the machine learning system has to learn images from the target altitude in advance. Mondragon *et al.* [10] proposed a stereo vision attitude-detection system for helicopter applications, and Song *et al.* [11] proposed a robust downward-looking method that uses two cameras to estimate the distance between the camera and the terrain. In the proposed method [10,11], the baseline of the two cameras is fixed and is shorter for aircraft flying at lower altitudes; however, stereo cameras cost more than a single camera. Milella *et al.* [12] proposed a multi-baseline stereo vision system for accurate scene reconstruction and segmentation at a wide range of viewing distances. The multi-camera system can be applied at various distances with high estimation accuracy. However, the multiple cameras required by the multi-baseline method again incur a cost and payload that is higher than that of a single camera.

This paper proposes an altitude detection system that utilizes a single camera and the velocity detected by the global positioning system (GPS) to simulate a stereo-vision system and estimate the length of the baseline. In this paper, two successive images are treated as stereo-vision image pairs from which to find disparities. However, the effect of wind means that the baseline of each image pair is not fixed. Wind changes the flying direction and velocity of UAVs. Thus, this study proposes flying direction detection and altitude calculation algorithms. Because the velocity of UAVs or the sampling rate of image frames can change the length of the baseline, the proposed system controls the baseline length with a single camera. For verification, the proposed algorithms are integrated into a radio-controlled aircraft that attempts to maintain a fixed altitude. On the flight path, it is necessary to avoid obstacles. Marlow and Langelaan [13] proposed optical flow measurement to compute and estimate the range of obstacles through an unsurveyed environment. In a previous paper [14], we proposed an algorithm for avoiding obstacles that appear in the flight path in the intermediate region of the image. In this paper, we use this previous method to achieve a collision-free flight path through which the aircraft can pass without encountering obstacles. Consequently, the problem of obstacle avoidance is not discussed in this paper. 

In this study, a forward-looking camera, downward-looking camera, and electronic equipment are assembled on a radio-controlled aircraft. The forward- and downward-looking cameras individually perform the function of different monocular vision detection. A forward-looking camera is installed to facilitate stable flight in conjunction with the skyline detection algorithm [15]. A downward-looking camera is also installed to capture continuous images with the use of the stereo vision algorithm, enabling the detection of the distance between the aircraft and ground. Altitude information from GPS cannot maintain a UAV at a fixed altitude from the ground, but image processing can. The downward-looking camera captures two consecutive images that are processed by the stereo vision algorithm to obtain the altitude between the aircraft and the ground. To obtain high-resolution images, the UAV must fly stably at low altitude. The ground images captured by the downward-looking camera at the fixed low altitude can capture images at the same and good ground image resolution at different terrain.

In this study, our objective is to explore vision sensors through an analysis of image sequence processing by the monocular camera in steady flight. The aim is to provide an alternative that reduces the cost of UAVs and recovers terrain information via monocular cameras. The remainder of this paper is organized as follows. Section 2 outlines the design and integration of the proposed UAV system, and Section 3 discusses flying direction detection and the relative altitude calculation and maintenance of the UAV flight system. In Section 4, we present experimental results from a fixed altitude flight. Finally, Section 5 outlines our future development plans.

## 2. Design and Integration of the Proposed UAV System

**Figure 1:**
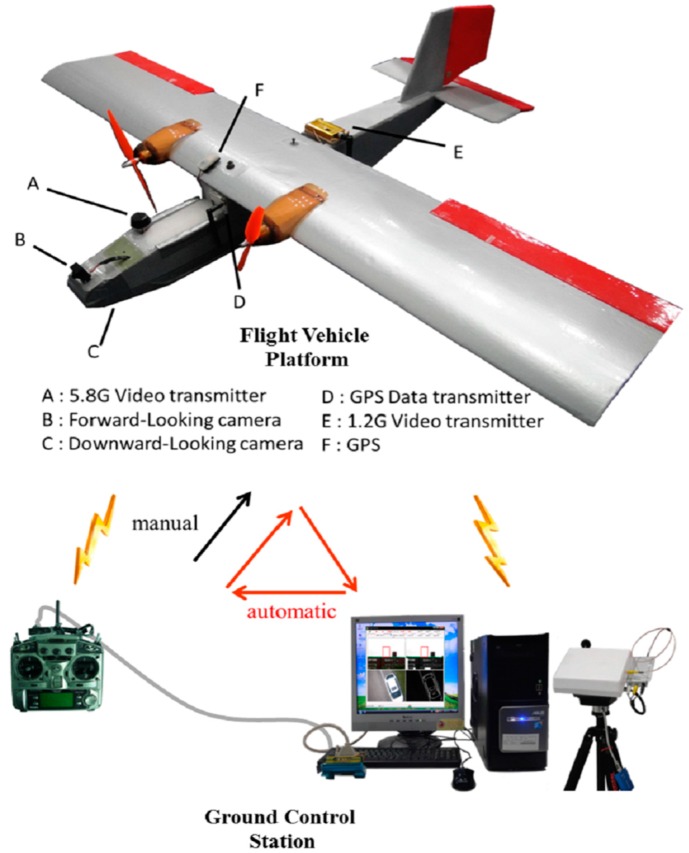
Architecture of the UAV FCS.

This section introduces the architecture of the flight control system (FCS) utilized for automatic flight (Figure 1). The FCS hardware can be divided into the flight vehicle platform (FVP) and ground control station (GCS). The FVP is equipped with two video transmitters operating on different frequencies, a forward-looking camera, downward-looking camera, and GPS data transmitter. The videos obtained from the forward- and downward-looking cameras are transmitted by different transmitters to video receivers at the GCS. The control signal of the remote controller and the image signal are transmitted in the form of an electromagnetic wave. The velocity of electromagnetic wave is 3 × 10^8^ m/s. When the distance between UAV and GCS is 200 m, the transmission time of electromagnetic wave is approximately 0.67 × 10^−6^ s. Thus, the communication latency of the control and video signals can be ignored. This study uses video transmitters on 1.2 GHz and 5.8 GHz frequencies to avoid wireless interference. The video is then converted to digital images by a USB video capture card. The images captured from the forward-looking camera are transmitted to the GCS computer, which is programmed to detect the skyline. The aircraft carries out a stable automatic flight with the aid of the skyline-detection algorithm [16]. The continuous images captured from the downward-looking camera in stable flight are also transmitted to the GCS computer. Two consecutive images from the downward-looking camera are treated as images captured by two cameras, as in a stereo vision system. Finally, the altitude between the aircraft and the ground is calculated by the proposed algorithm. Details of the FVP, GCS, and remote-control operation are described in the following sub-sections.

### 2.1. Specifications of the Flight Vehicle Platform 

The FVP comprises an airframe, two cameras (Sony charge-coupled device (CCD) 1/3 inch), a servo control receiver module, GPS and GPS data transmitter module, two video transmitter modules, and a power supply module. We designed and constructed a battery-powered double-motor aircraft (airframe length: 108 cm) with wide wings (wingspan: 150 cm) and four channel servos (controlling rudder, ailerons, elevator, and throttle). The forward-looking camera is installed on the edge of the front airframe, and the downward-looking camera is mounted at the lower edge of the front airframe. Video transmitters send the images captured by the two cameras at different frequencies (1.2 GHz, 5.8 GHz) to the GCS video receivers. The signal from the remote control (RC) is sent by an RC receiver that controls the direction, posture, and altitude of the UAV. The GPS obtains information (longitude, latitude, horizontal altitude, and velocity) from satellites, and transmits it to the GPS receiver module of the GCS.

### 2.2. Specifications of the Ground Control Station 

The GCS comprises a computer platform, antenna tracking pan-tilt-zoom (PTZ), two video receivers, a GPS receiver, and an RC transmitter. The tracking PTZ can track the position of the UAV via the signal from the tracking transmitter of the FVP to obtain maximum received radio power. The tracking PTZ is able to rotate 360° with video receivers to receive clear images from the video transmitters of the FVP. After receiving two video signals, the USB video capture card transfers the video signals to digital image sequences in the GCS computer. During the operation of the aircraft control program, a digital-to-analog (D/A) converter converts the digital control signals, including the roll and pitch angles, to the analog signals used by the RC. The analog signals are then transmitted to the UAV by the RC, which controls the roll and pitch angles of the UAV. The computer is equipped with an Intel Core i7-4790 (3.6 GHz) processor and 16 GB RAM. In this study, the average processing time for skyline detection and altitude detection is 1 ms and 16 ms, respectively.

### 2.3. Manual/Automatic Remote Controller

**Figure 2:**
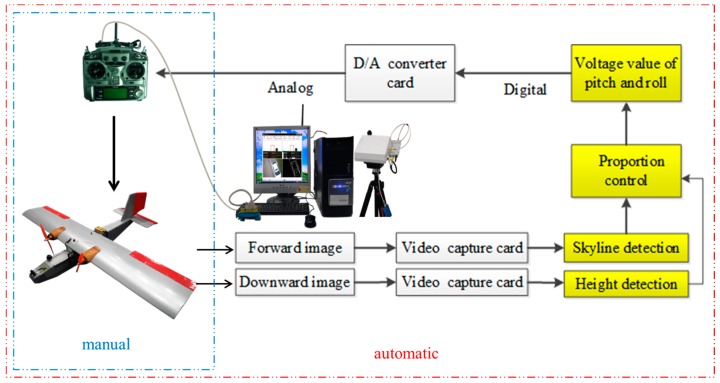
Manual/automatic FCS architecture.

Digital control signals are transformed by the detection results of the skyline detection and relative altitude detection algorithms. These digital control signals are subsequently converted to the analog control voltage of the RC by the D/A converter. A switch is installed on the RC to switch between manual and automatic flight modes. In manual flight mode of the RC, an operator controls the takeoff and landing of the aircraft, whereas in automatic flight mode, the FVP transmits real-time images to the GCS while executing skyline detection and relative altitude detection to analyze the control values for the pitch and roll angles. On determining the control value for the attitude, it can change the voltage signal using a proportional controller. The RC is controlled by the computer to modify the flight attitude so as to maintain the predefined relative altitude and ensure horizontal flight stability. The architecture of the manual/automatic flight system is depicted in Figure 2.

## 3. Relative Altitude Maintenance Flight System

As stated above, the UAV has forward- and downward-looking cameras that are used to capture images and detect the skyline and relative altitude, respectively. The results from the image detection algorithms are transformed to voltage signals, and these are used by the RC to control the servo of the UAV. The roll servo of the UAV is controlled by the detected roll angle of the skyline detection algorithm to keep the flight stable, and the pitch servo of the UAV is controlled by the relative altitude detection algorithm to maintain a fixed altitude between the aircraft and the ground. Above smooth ground, major attitude change is caused by the skyline detection algorithm. Because the detection rate of the skyline detection is 30 images/s, the roll angle of the UAV changes about 1/30 s. According to the experimental results, the variation of the roll angle is within 5 degrees/s. Therefore, the attitude change caused by the roll angle is around 0.167°. When the distance between the UAV and ground is 30 m, the bias caused by the attitude change is around 0.087 m on the ground. Because the bias is very small, the attitude change caused by the roll angle can be neglected. The relative altitude maintenance procedure can be separated into three stages: (1) flying direction detection, to find the flying direction from the ground images; (2) relative altitude calculation, to estimate the relative altitude according to the computed baseline given by the GPS information on the aircraft; and (3) relative altitude maintenance, to control the pitch angle of the UAV so as to maintain a fixed altitude. In this study, the roll angle is controlled by the skyline detection algorithm to stabilize the roll angle of the UAV; the pitch angle is controlled by the altitude detected by the flying direction detection algorithm and relative altitude calculation methods to control the relative altitude. Therefore, these methods are performed independently. Details of these three stages are described in the following subsections.

### 3.1. Flying Direction Detection

A single camera solution is proposed to detect the relative altitude. Based on the single camera system, stereo image pairs are matched from the continuous image sequence. Therefore, the baseline is determined by the distance of the neighbor images. The distance between neighboring images is influenced by the flying direction and the velocity of the UAV. The direction of flight is given by the flying direction detection algorithm, and the velocity is provided by GPS information.

**Figure 3:**
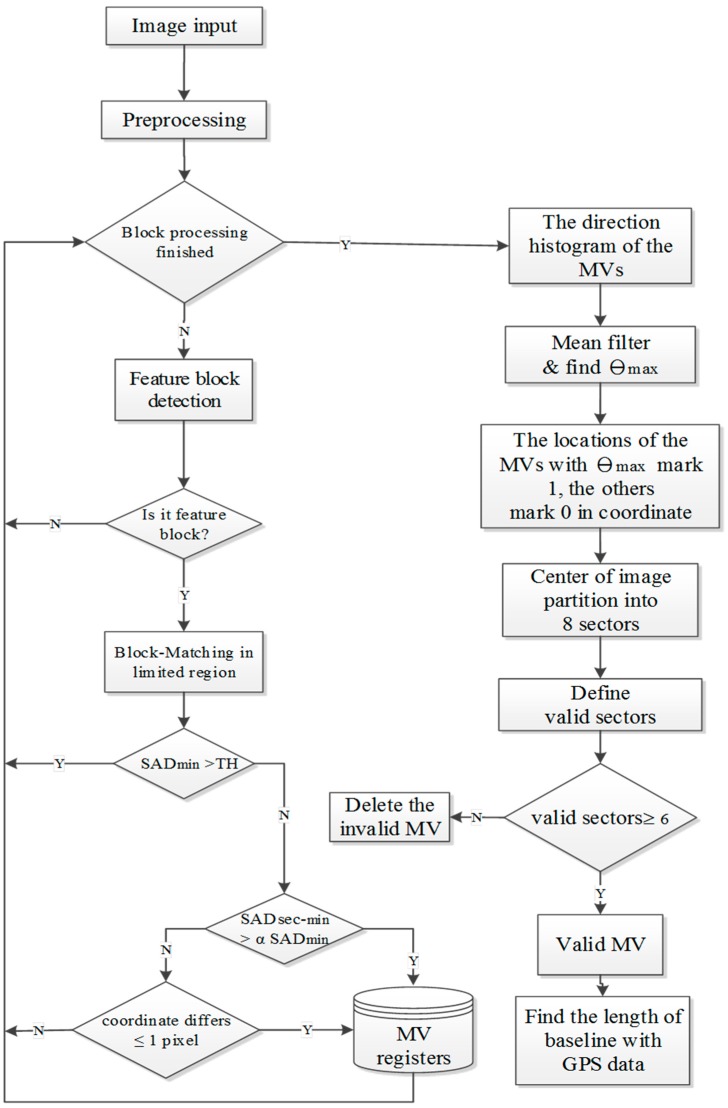
Flying direction detection algorithm.

In this study, we use the downward camera to capture ground images and detect the direction of flight with respect to the ground. A flowchart of the flying direction detection algorithm is shown in Figure 3. First, in the preprocessing phase, the downward-looking camera captures ground images of 480 ×640 pixel resolution. The National Television System Committee (NTSC) format of video signals means that the captured images suffer from the interlace problem, which influences image recognition. Therefore, the captured images must be de-interlaced to 240 × 640 pixels. The processed images are transformed to gray images, and are then segmented into non-overlapping blocks. In general, the reliability of the block-matching result is proportional to the block size. Considering the reliability of the block-matching result and the number of blocks, the block size is chosen to be 32 × 32 pixels.

In the feature block selection shown in Figure 3, segmented blocks with sufficient edge points (detected by 3 × 3 Sobel edge detection) are selected as feature blocks. Feature blocks with more edge points signify higher complexity in the image blocks. In general, these feature blocks can obtain better matching results than non-feature blocks under the block matching method. Therefore, non-feature blocks are bypassed in the following process. The threshold for detecting feature blocks is set to 30 edge points. This threshold value is chosen by conducting experiments under different environments. When the number of edge points within a block is greater than 30, the block is selected as a feature block.

**Figure 4:**
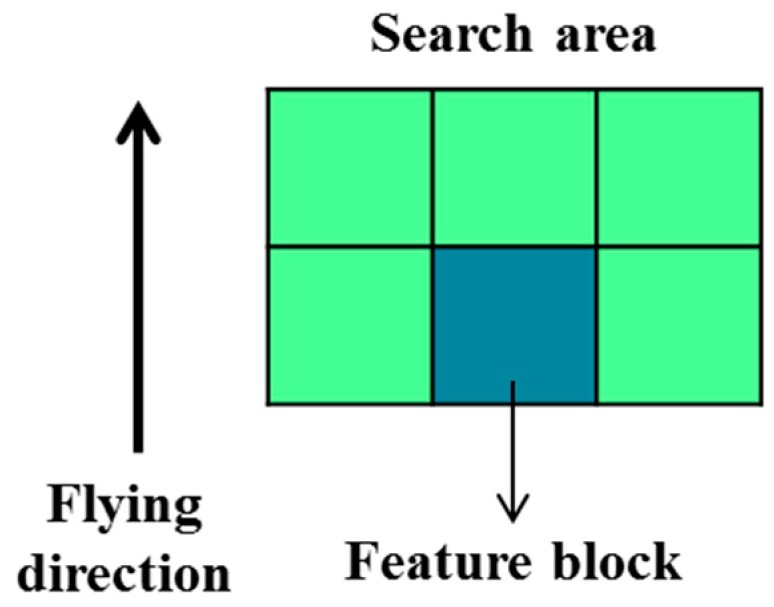
Search area of full-search block matching method.

After feature block selection, the feature blocks are processed using the full-search block matching method with the previous image. Because the motion vector is around 18 pixels when the speed of UAV is 40 km/h, the search area of the block matching method is 96 × 64 pixels. The search area is shown in Figure 4; this study assumes the direction of movement of the UAV to be forward. 

Feature block selection is the first step in decreasing the matching error between two continuous images. Because the block matching method searches for locations with the minimum sum of absolute difference (SAD_min_) as the best match within the search area, the search results of feature blocks may have erroneous matches. To filter out these results, two cases are classified as untrustworthy matches in the second step. The first case is when the SAD_min_ value of the block matching method is not small enough. This suggests the two block images are not similar to each other. The second untrustworthy match type is when the secondary minimum sum of absolute difference (SAD_sec-min_) value of the block matching method is close to the SAD_min_ value of the block matching method. This indicates that the representative of the SAD_min_ value is not good enough. One exception to the second case occurs when the location of the SAD_sec-min_ value is close to the location of the SAD_min_ value. Such similarities could exist when the position of the best match is located on the range between two pixels. In the first case, we set a threshold, TH = 32 × 32 × ε, to filter out the untrustworthy matches when SAD_min_ is greater than TH. Because the block size is 32 × 32 pixels in this study, ε is the acceptable error of each pixel, which is set as five. Because the flying altitude is set to be low in this study, the occluded or missing problems caused by trees or buildings will result in error matches. Therefore, the feature blocks are deleted when the SAD_min_ value in the block matching method is greater than TH. For the second case, we filter feature blocks for which the SAD_sec-min_ value is similar to the SAD_min_, but that are not located close to the minimum-value feature block. The parameter α is set to 2. The parameter is chosen by conducting experiments under different environments. The differences of coordinates between SAD_min_ and SAD_sec-min_ is calculated, which is less than one pixel or equal to one pixel, it can be retained in the motion vector (MV) register. Otherwise, it can be deleted.

After the block matching and filtering processes, the motion vectors (MVs) of the trusted feature blocks are processed to find the valid motion direction/flying direction. The directions of the motion vectors represent the flying direction relative to the ground. Because of limitations in the pixel resolution and the undulating ground, the directions of the motion vectors have a centralized distribution. The histogram of motion vector directions is processed by a mean filter with five neighbor histogram values to smooth the direction histogram. After processing the mean filter, the angle with the largest number in the direction histogram is labeled as θ_max_. The positions of the motion vectors with the same angle as θ_max_ are marked as one; the others are marked as zero. The four quadrants are each partitioned into two sectors of 45°. For each sector, we count the total number of motion vectors with the same angle as θ_max_. When the count in any sector is at least two, that sector is defined as a valid sector. When more than six sectors are valid, the angle θ_max_ is considered to be a valid flying direction; otherwise, the flying direction detection is invalid for that image pair. Invalid detections in which the feature blocks of θ_max_ do not have a widespread distribution are deleted to prevent errors caused by moving objects on the ground.

### 3.2. Relative Altitude Calculation

**Figure 5:**
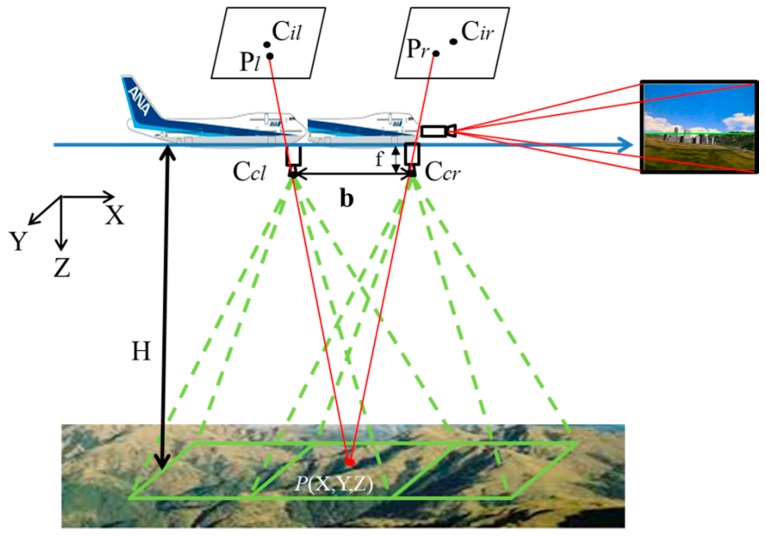
Relative altitude calculation with a single camera.

After the flight direction has been determined, changes in direction caused by wind can be detected. Hence, the direction of the baseline can be detected from the flying direction. When the length of the baseline is estimated, the altitude between the UAV and the ground can be calculated. Figure 5 illustrates how monocular vision-based altitude measurement is carried out for UAVs. The forward-looking camera captures images that are then processed by the skyline detection algorithm to maintain aircraft stability. The downward-looking camera captures two consecutive images that are processed by the stereo vision algorithm to obtain the relative distance between the airplane and ground. Wang *et al.* [16] and Song *et al.* [11] calculated the altitude information using two cameras. In this study, only one camera is used to detect the relative altitude. Assume that a reference point *P* is located on the ground. Figure 5 illustrates the detection of the altitude between the aircraft and *P*. Assume the length of the baseline *b* is as shown in Figure 5, and that the parameter *f* is the focal length of each camera. *C_il_* and *C_ir_* are the centers of consecutive images. The reference point *P* is projected to the consecutive images to give *P_l_* and *P_r_*, respectively. Along the direction of the baseline, assume the projection value of $\left(P_{l}-C_{il}\right)$ is Δ*d_l_* and the projection value of $\left(P_{r}-C_{ir}\right)$ is Δ*d_r_*. The parameter *W_pixel_* is the width in pixels of the image sensor, and the parameter *H* is the distance between the center point of the baseline and *P*.

Using the geometric principles of similar triangles, the distance between the center point of the baseline and the reference point *P* can be computed as:

(1) $$H=\frac{f\times b}{\left(\Delta d_{l}-\Delta d_{r}\right)\times W_{pixel}}$$

For the block matching method with two consecutive images, the magnitude of the motion vector along the flying direction, which is the same as the direction of the baseline, is the disparity Δ*d*:

(2) $$\Delta d=\Delta d_{l}-\Delta d_{r}$$

Therefore, the relative altitude can be expressed as:

(3) $$H=\frac{f\times b}{\Delta d\times W_{pixel}}$$

**Figure 6:**
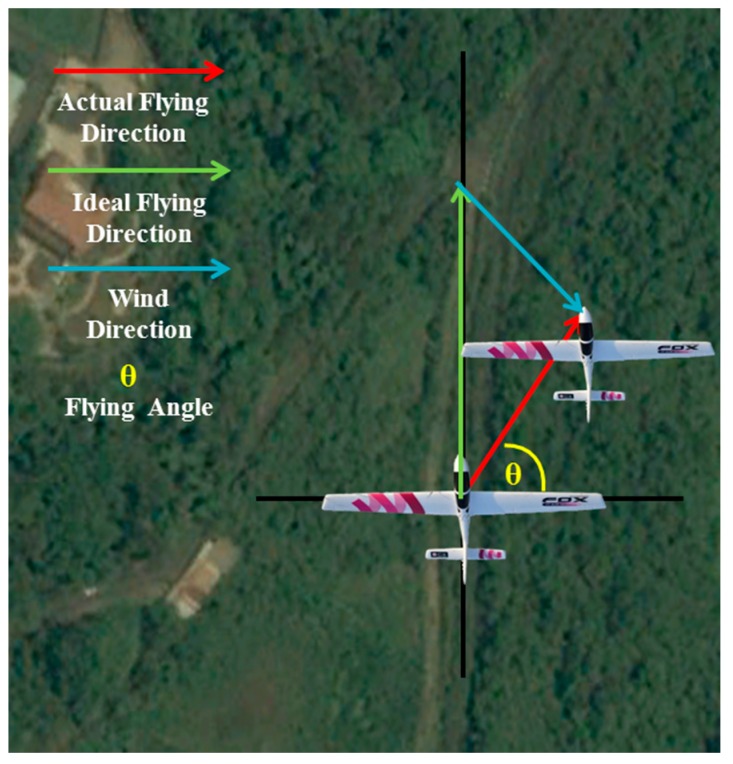
Flying direction changed by wind.

The value Δ*d* detected by the flying direction detection process is the magnitude of the motion vector along the direction of flight. The last unknown parameter is the baseline *b*. Because the wind could affect the flying direction and velocity, as shown in Figure 6, distance estimation between consecutive images is a difficult task. 

Thus far, the flying direction and the magnitude of the motion vector along the flying direction are given by the proposed algorithm. The time difference between consecutive images can also be recorded during the capture process. The final variable is the velocity of the UAV. Therefore, a GPS module is added to the UAV to provide velocity information. In this study, the GCS obtains the velocity value from the GPS transmitter module at 10 Hz. This study uses Vincenty’s formulas [17] to calculate the distance between two GPS points and the velocity following the GPS fluctuation. Because the frame rate is 30 frames per second (30 Hz), the velocity data cannot be updated with each image frame. Fortunately, the velocity changes only slightly in 1/30 of a second, and so is assumed to be constant over this period. The length of the baseline is then calculated from the velocity and the time difference between the two images. The relative altitude is calculated by Equation (3).

### 3.3. Relative Altitude Maintenance

In the proposed system, the forward-looking camera is used for skyline detection, which helps maintain the stability of the aircraft in flight. The downward-looking camera continuously captures images to detect the relative altitude between the aircraft and the ground. The relative altitude is used to maintain a fixed altitude during the flight. As discussed previously, the detection results effectively modify the voltage to the RC to maintain a fixed altitude. We define FH as the predefined altitude of the flying mission, DH as the detected altitude of the proposed algorithm, and VRC as the voltage of the RC. The minimum/maximum voltage of the pitch control of the RC is 0.004/4.996 V, and the intermediate voltage is 2.5 V. Let ∆H denote the deviation from FH. A negative value signifies that the flight altitude of the UAV is higher than FH, and vice versa. To lower the altitude of the UAV, the voltage of the pitch control has to be reduced. The FCS control values in terms of ∆H and VRC are displayed in Table 1.

**Table 1:** Altitude-Voltage table.

∆H = FH − DH (m)	V_RC_ = Voltage of RC (volts)
−10 < ∆H < 0	V_RC_ = ⌊∆H/2⌋×0.416+2.5
∆H < −10	V_RC_ = 0.004
0 < ∆H < 10	V_RC_ = ⌈∆H/2⌉×0.416+2.5
10 < ∆H	V_RC_ = 4.996
∆H = 0	V_RC_ = 2.5

## 4. Experimental Results

**Figure 7:**
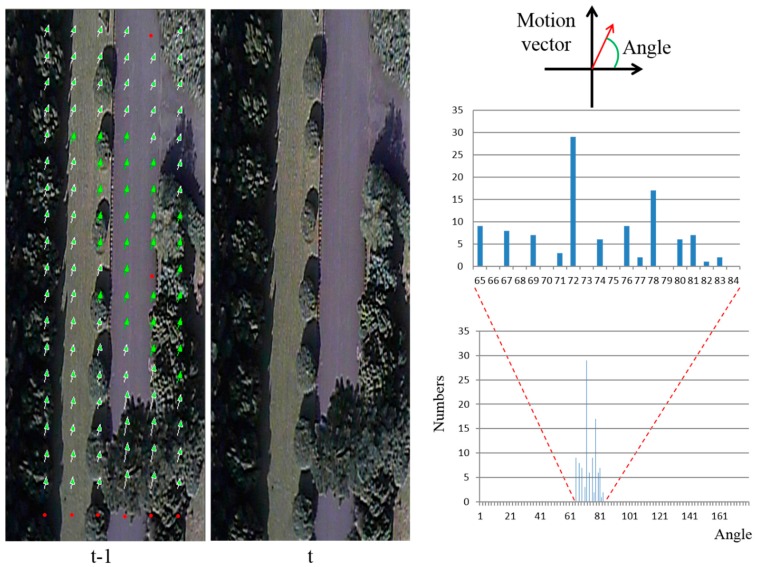
Detected results of the motion vectors.

This section presents and discusses the experimental results obtained for flying direction detection, relative altitude calculation, and relative altitude maintenance. The experimental results prove that the algorithm for flying direction detection is accurate and practical. The detected altitude between the UAV and the ground is transferred to the control voltage of the RC to maintain a fixed altitude of flight. Figure 7 shows the motion vectors detected by the flying direction detection, and the histogram of motion vectors for two consecutive images.

**Figure 8:**
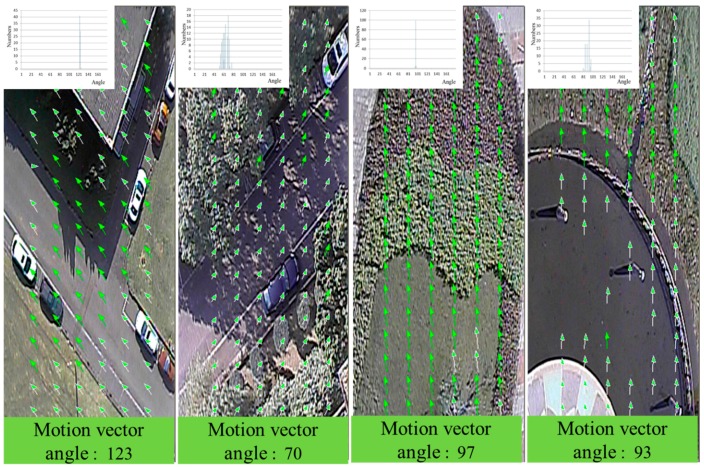
Different detection results under various terrains.

On the left of Figure 7, the valid flying direction, *i.e.*, the same angle as θ_max_ = 72°, is marked as green arrows. There are 29 such motion vectors. The other motion vectors are marked as light green arrows. From the detection results, it is obvious that the proposed flying direction detection algorithm is suitable for detecting the motion between consecutive images. The valid flying direction of 72° indicates that the influence of the wind is pushing the UAV to the right by 18°. The magnitude of the motion vector is the disparity of the stereo vision. The larger the magnitude of the motion vector, the closer the ground is to the UAV. Therefore, the smallest magnitude motion vectors signify the distance between the UAV and the ground, rather than buildings or trees. Figure 8 illustrates different detection results for various terrains.

In Figure 7 and Figure 8, the red points denote invalid feature blocks. We can see that these invalid feature blocks are located on smooth regions in the image. These blocks are not suitable for detecting motion vectors. From the detection results, it is clear that the proposed detection method can successfully detect invalid feature blocks. In Figure 8d, the blue point marks a location at which the SAD_min_ value of the block matching method is not small enough; this means that the matching result is poor, and the comparison result is deleted.

**Figure 9:**
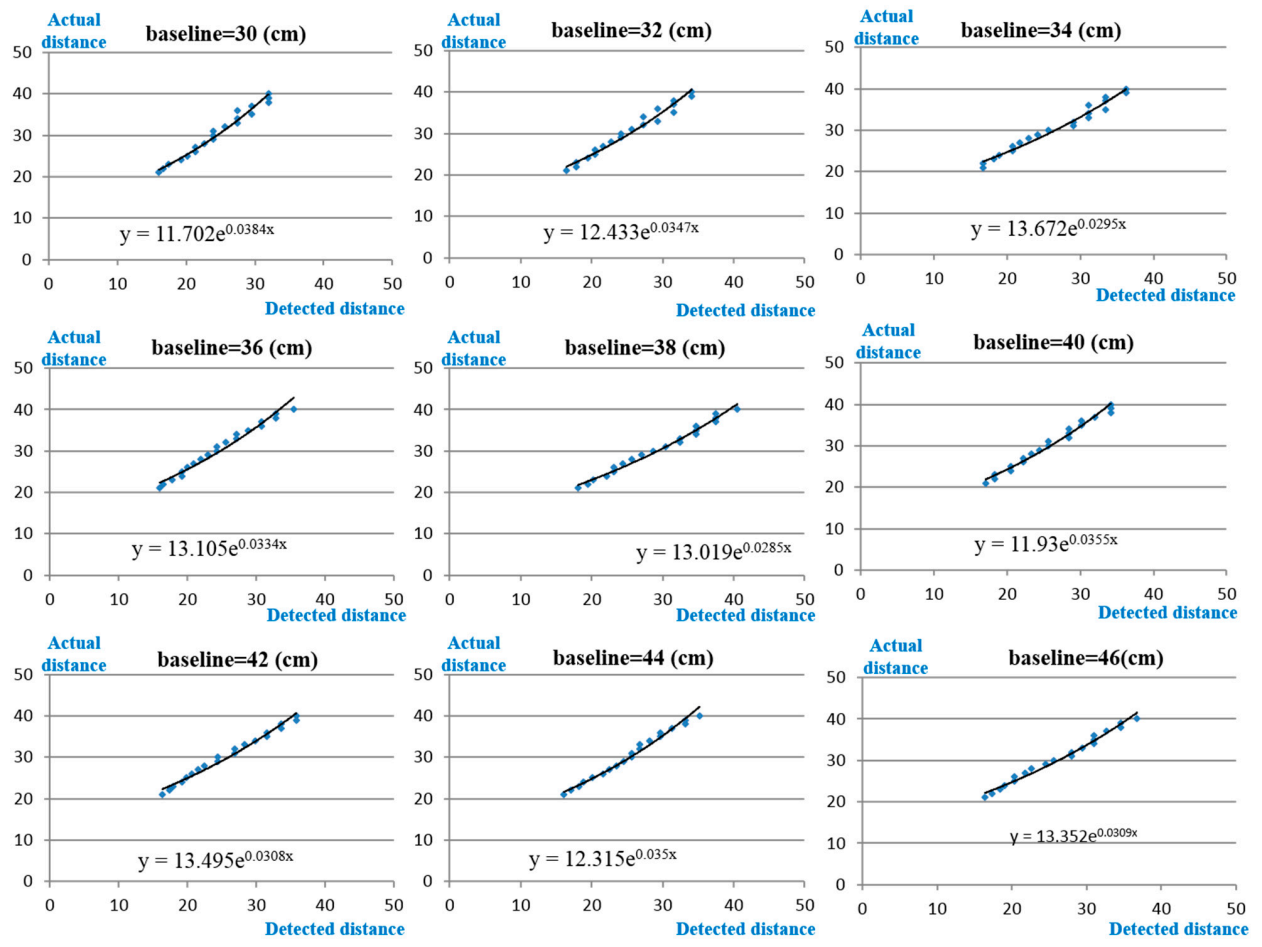
Partial calibration results under different baselines.

Because the real altitudes during these experiments are very difficult to measure, an error analysis of the relative altitude calculation is provided to decrease the calculation error. Because the camera and lens are low-priced products, the captured images have a slight deformation. This image deformation causes detection errors in the distance estimation. The image deformation problem can be calibrated by the image calibration method or the calibration table. Because this study considers the problem of the execution speed, the calibration table is used to correct the measurement errors. During calibration, the same camera and lens are used to capture two images with different baselines, and the measuring targets are placed between 20 m and 40 m from the camera system. The baselines are varied every 2 cm and they range from 20 cm to 50 cm. Figure 9 presents the partial calibration results of the measurements. The *x* coordinates of the calibration tables represent the detected distance, which is estimated by the image detection algorithm, and the *y* coordinates of the calibration tables represent the actual distance. When the detected distance is obtained by the proposed system, the detected distance is the *x* coordinates to find the corresponding value of the *y* coordinates with the closest baseline.

**Figure 10:**
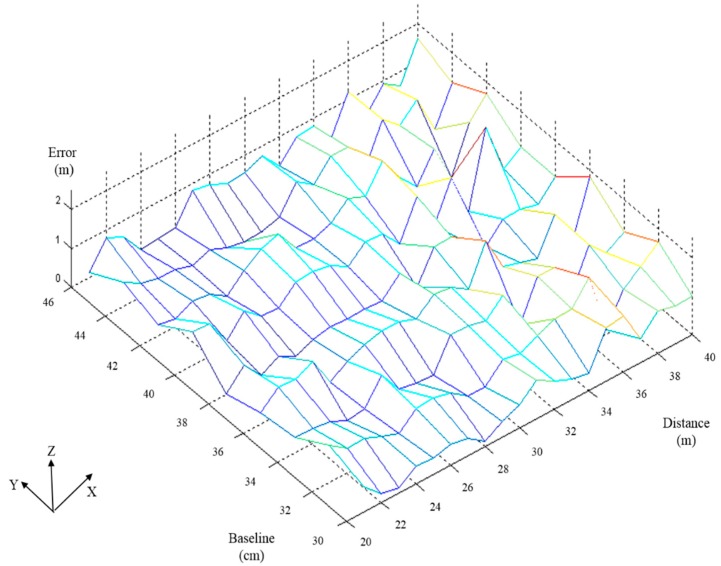
Error analysis of the calibration tables shown in Figure 9.

Figure 10 shows the error analysis of the relative distance between the detected distance and real distance. In this figure, *X*-axis represents the real distance varying from 20 m to 40 m, *Y*-axis represents the baseline varying from 30 cm to 46 cm, and *Z*-axis represents the error between the detected distance and the real distance. The error values are within 2 m in Figure 10.

The test site includes an amphitheater. The seating area of the amphitheater is located on the hillside, and the drop height of the sitting area is around 18 m. In the flying test, the field of view is vast. Data are received from a total of eight GPS satellites, which implies that accurate velocity data are obtained from the GPS system. The wind speed on the ground is around 0–1.3 m/s, and is very different at various altitudes. The time period of the flying test is 12.3 s, and the average speed of the UAV is 39.6 km/h. The actual flight distance is 135.3 m, over which a total of 370 ground images are recorded.

**Figure 11:**
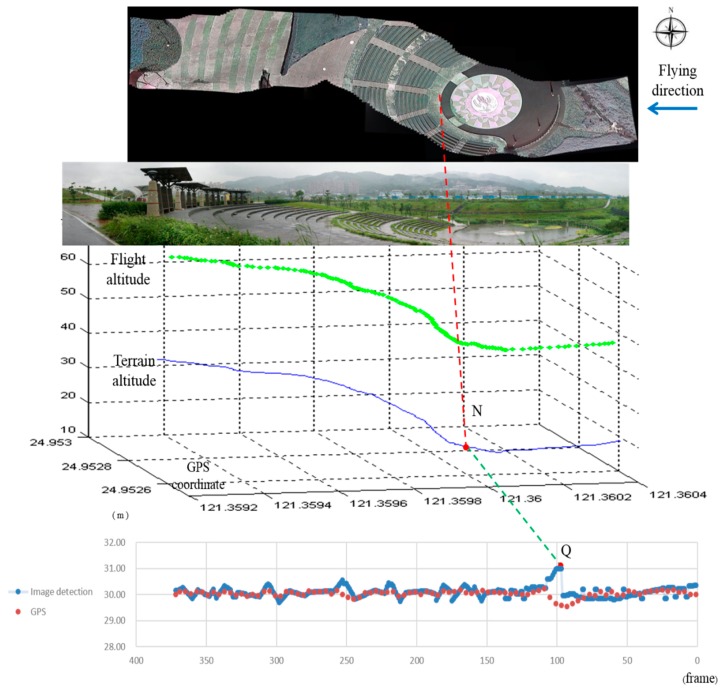
Experimental results from the proposed system.

Figure 11 shows the test site and the experimental results of the flying test. The flying direction of the UAV is from east to west. From top to bottom, Figure 11 shows the combined image with image stitching, a photo of the test site, the terrain altitude, and the detected relative altitude. The combined image in Figure 11 combines the 370 images using photo stitching software to show the flight path. The combined image is misaligned, because the flying direction is influenced by the cross-wind.

In the terrain altitude figure, the horizontal plane is the GPS coordinate and the vertical-axis is the elevation. The blue line in this figure is the terrain altitude, which is measured by carrying a GPS and walking underneath the flight path on the ground. The chart at the bottom of Figure 10 shows the detection results for the altitude between the UAV and the ground, as detected by the proposed algorithm. In this figure, the *x*-axis is the frame number and the *y*-axis is the variation in the detected relative altitude. There is one discontinuous point Q that is caused by the UAV suddenly climbing. Point N in the terrain altitude figure corresponds to Q. N is the point at which the pitch of the UAV starts to pull up, resulting in a steep change in relative altitude. Therefore, the pitch of the UAV changes suddenly to maintain the predefined relative altitude. The detected relative altitude of point Q is 30.99 m. The difference between the predefined relative altitude and detected relative altitude is transferred to a voltage signal to the RC that adjusts the flying altitude. Because this study focuses on the detection algorithm, the simple control method used results in the UAV swinging up and down. However, the deviation between the predefined altitude and the detected altitude remains within 1 m. 

The relative altitude can be computed by the GPS signals of the UAV and the ground. In the flying test, the GPS data and the ground images are transmitted from the UAV to the ground control station. The average flying speed of the UAV is around 39.6 km/h and the flying time is 12.3 s. The data rate of the GPS is between seven to eight times per second. The number of the GPS data packets for recording the flying path of the UAV is 93. The ground images transmitted by the UAV are combined using an image stitching software to indicate the ground path relative to the flying path of the UAV. After the landing of the UAV, the GPS system is used to record the ground altitude by walking along the ground path shown in the combined image. The number of the GPS data packets is 794. We use the positions of the UAV and the ground to compare the altitude of GPS data. The relative altitudes computed using the two GPS data are shown in Figure 11. The green points in this figure indicate the flying path of the UAV.

This study proposes a vision detection system to maintain a fixed relative altitude flight for the UAVs. Because the pitch angle of the UAVs change based on the terrain, the attitude will be changed. However, the geometry shown in Figure 5 is valid in this study because the UAV is still parallel to the ground surface. The experimental results for relative altitude maintenance integrate images from the forward-looking camera to keep the flight stable using the skyline algorithm and the downward-looking camera to maintain a predefined relative altitude using the proposed algorithm. Therefore, the proposed system can maintain a low attitude with respect to the terrain, thus obtaining better image resolution.

## 5. Discussion and Conclusions

In this paper, we have described a system in which a UAV is fitted with forward- and downward-looking cameras that are used to maintain a stable and predefined altitude in conjunction with skyline and relative altitude detection algorithms. The length of the baseline is calculated from the flight speed, which is provided by GPS data. In the flight test, eight GPS satellite signals were received. The deviation between the predefined altitude and the detected altitude was less than 1 m. The skyline detection and relative altitude detection algorithms required around 1 ms and 16 ms processing time on an Intel Core i7-4790 processor with 16 GB RAM. The proposed system not only obtains a consistent image resolution with respect to the terrain but also maintains a constant altitude and stable flight. In this study, the flight path is influenced by the cross-wind. Therefore, the future work will add a straight keeping detection algorithm to maintain a straight flight. Moreover, the monocular vision system for terrain tracking will also be used in future work. In addition, ground images can be tracked continuously to analyze the yaw angle of the UAV. The yaw angle and the flying direction can be used as a navigation system to control the UAV during GPS signal loss.
